# Systematic analysis of the effect of multiple templates on the accuracy of comparative models of protein structure

**DOI:** 10.1186/1472-6807-8-31

**Published:** 2008-07-16

**Authors:** Suvobrata Chakravarty, Sucheta Godbole, Bing Zhang, Seth Berger, Roberto Sanchez

**Affiliations:** 1 Department of Structural and Chemical Biology, Mount Sinai School of Medicine, 1425 Madison Avenue, New York, NY 10029, USA

## Abstract

**Background:**

Although multiple templates are frequently used in comparative modeling, the effect of inclusion of additional template(s) on model accuracy (when compared to that of corresponding single-template based models) is not clear. To address this, we systematically analyze two-template models, the simplest case of multiple-template modeling. For an existing target-template pair (single-template modeling), a two-template based model of the target sequence is constructed by including an additional template without changing the original alignment to measure the effect of the second template on model accuracy.

**Results:**

Even though in a large number of cases a two-template model showed higher accuracy than the corresponding one-template model, over the entire dataset only a marginal improvement was observed on average, as there were many cases where no change or the reverse change was observed. The increase in accuracy due to the structural complementarity of the templates increases at higher alignment accuracies. The combination of templates showing the highest potential for improvement is that where both templates share similar and low (less than 30%) sequence identity with the target, as well as low sequence identity with each other. The structural similarity between the templates also helps in identifying template combinations having a higher chance of resulting in an improved model.

**Conclusion:**

Inclusion of additional template(s) does not necessarily improve model quality, but there are distinct combinations of the two templates, which can be selected *a priori*, that tend to show improvement in model quality over the single template model. The benefit derived from the structural complementarity is dependent on the accuracy of the modeling alignment. The study helps to explain the observation that a careful selection of templates together with an accurate target:template alignment are necessary to the benefit from using multiple templates in comparative modeling and provides guidelines to maximize the benefit from using multiple templates. This enables formulation of simple template selection rules to rank targets of a protein family in the context of structural genomics.

## Background

Comparative modeling uses experimentally determined protein structures (templates) to predict the 3D conformation of another protein with a similar amino acid sequence (target). With the progress of structural genomics initiatives, comparative (or homology) modeling has become an increasingly important method for building protein structure models [1-3]. Not only is comparative modeling the most accurate method of structure prediction [4], but it also allows *a priori *estimation of the approximate quality of the models [5]. Due to their added value [6], models are particularly suitable for comparative studies over complete protein families [7-9]. However, predicted structures in general contain errors and seldom reach the accuracy of experimental structures. Hence, improving the quality of comparative models, especially for models where Target:Template sequence identity is less than 30% still remains a challenge [10].

Three elements that influence the accuracy of comparative models [11] are: (i) the structural similarity between target and template, (ii) the Target:Template alignment accuracy; and (iii) our ability to refine the model (i.e. loop modeling and general refinement). Hence, quality (measured as errors) of a model in terms of these factors can be described as:

(1) **Total Error = Structural Difference + Alignment Error - Refinement**

**Figure 1:**
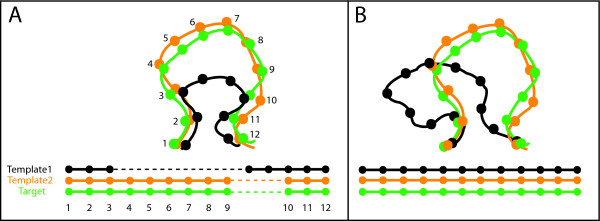
**Structural complementarity of templates**. **(A) **Absence of structural information from Template1 for segment involving residues 4–9 in the target is complemented by an equivalent segment in Template2. **(B) **A segment of the Target can be structurally closer to Template2 than Template1. Template1 refers to the template with the higher sequence identity (see Methods).

Significant effort has been devoted to the development of methods for refinement of models, but to date no protocol has emerged that systematically and predictably improves the quality of comparative models [12,13]. Advances in sequence alignment methods have proven successful in improving model accuracy by decreasing the alignment error (**Eqn. 1**). Most recently, the use of profile-to-profile alignments [14-16] has shown promise. Controlling the structural difference component of the error (**Eqn.1**) to improve model accuracy translates into the problem of selecting the template that is structurally closest to the target. In real modeling cases, the structure of the target is unknown and the structural difference between target and template must be estimated by other means. Usually, the template with the highest (or statistically most significant) sequence similarity is chosen. In spite of some evidences to the contrary [17], a number of modeling examples have shown that it is possible to improve model accuracy by simultaneously using more than one template structure [4,18-20]. It is expected that the best combination of all available templates needs to be chosen for multiple template modeling. The best possible combination of templates should in principle be that where segments of different templates constitute a template chimera that is structurally closer to the target than any of the individual templates. There are two commonly observed template combinations (chimera) where one template structurally complements the other. These are: (i) absence of structural information in one template can be complemented by a second template (Figure 1A); (ii) a segment of the target that shows low structural similarity with one of the templates may show higher structural similarity with the second template (Figure 1B). We refer to these as **structural complementarity of the templates**. Anecdotal evidence indicates that structural complementarity may result in improved model accuracy [18], but so far no systematic study has been performed to assess the effect of multiple templates on the accuracy of comparative models in which the alignment and structural complementarity contributions are dissected. In this work, we address the effect of additional templates of lower sequence identity on the accuracy of the simple comparative models for which we previously characterized their accuracy and added-value [5,6]. To an existing Target:Template combination, another template of lower sequence identity is added. For example, Template2 (sequence-identity of S2% with the Target) is added to a Target:Template1 pair (sequence identity of S1% with the Target) such that S2 < S1. The model of the Target sequence based on Template1 (single-template) is compared to that based on the combination of Template1:Template2 (multiple-template) to understand the effect of addition of the second template, Template2. Any difference in the model quality in this experiment can be seen as a result of the variables described in **Eqn1 **(i.e. alignment error or structural difference). The alignment of Target:Template1 may be different from that of Target:Template1:Template2 and the observed change in the model quality could be due to the difference in the modeling alignment in the two situations. It could also be the case that the Template1:Template2 combination results in a template chimera that is structurally closer to the Target than each of the individual templates (see Figure 1) resulting in a better model without any changes to the alignments. It is also possible that a combination of both of the factors brings about changes in the model quality. In this study we seek to understand the contribution of structural complementarity in multiple template modeling. Hence, we assess the accuracy of multiple template models to answer the following questions: (i) On average, are multiple-template models more accurate than their single-template counterparts? (ii) What are the requirements to maximize the potential positive effect (improved model quality) of multiple templates on model accuracy? For clarity we answer these questions using the simplest case of multiple-template comparative model, namely two-template models.

## Results and discussion

### Research design

To facilitate the interpretation of the results the research design is described in this section.

Structure-based Target:Template alignments are the most accurate ones for structure modeling. While these structure-based alignments do not represent an alignment that is achievable in real modeling cases (because the target structure is by definition not known in these cases) they are a useful benchmark representing error free alignments. At the other end of the spectrum we find pairwise sequence alignments, which rely only on the knowledge of the sequence of the target and the template. Any difference in the quality of models based on these alignment types is solely due to differences in quality of the modeling alignment and has been the subject of our earlier study of single-template models [5]. STR and SEQ alignments used here correspond to these baseline alignments and are used to study the influence of alignment accuracy on multiple-template modeling accuracy.

**Figure 2:**
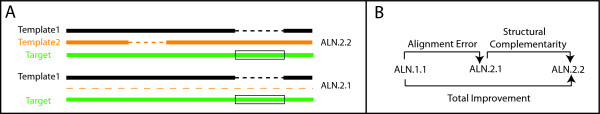
**Strategy to deconvolute the alignment accuracy and structural complementarity effects on two-template model accuracy**. **(A) **A pair of models is built alternatively on the same modeling alignment in presence of one (bottom) and both templates (top). The Target segment corresponding to the box has no structural information in absence of Template2. ALN stands for alignment type (SEQuence or STRucture). **(B) **The total improvement of multiple template models over single template models is a combination of decreasing alignment errors and structural complementarity.

For convenience, SEQ and STR single-template models in this study are named SEQ.1.1 and STR.1.1 respectively (See Table 1 and methods for a detailed description of the **ALN.X.Y **nomenclature used here) and the two-template models are named SEQ.2.2 and STR.2.2. ALN stands for alignment type SEQ or STR; and X refers to number of template sequences used to generate the modeling alignment and Y refers to number of template structures used in the actual modeling step after generating the modeling alignment. As discussed above (see Background), the difference between SEQ.1.1 and SEQ.2.2 can be a result of changes in the modeling alignment as well as structural complementarity of the first template by the second template (Eqn. 1, Figure 2B). To eliminate contributions from alignment changes, thus focusing the analysis on structural complementarity, we use the SEQ.2.1 models. The Target sequence in SEQ.2.1 is aligned simultaneously to two templates (X = 2) but the modeling step involves only one template, i.e. Template1 (Y = 1) (see methods). Thus a model accuracy difference that arises due to structural complementarity of the templates will be revealed by the comparison of ALN.2.1 vs. ALN.2.2 models (Figure 2B).

**Table 1:** Description of model types (ALN.X.Y nomenclature)

**Model**	**Alignment**	**Template**	**Description**
SEQ.2.2	Template1 and Template2 sequences are structurally aligned first. Target sequence is then aligned to both the templates sequence using a pairwise alignment algorithm without altering the structural alignment between the templates.	T1 & T2	A two-template model based on the simplest (least accurate) alignment. This model is influenced by the sequences and structures of both templates.
SEQ.2.1	Same as SEQ.2.2	T1	A one-template model based on the simplest (least accurate) alignment between target and both template sequences. This model is influenced by the sequences of both templates but only by the structure of T1.
STR.2.2	Target, Template1 and Template2 sequences are structurally aligned.	T1 & T2	A two-template model based on an error-free alignment derived from the structural superposition of the target, T1, and T2.
STR.2.1	Same as STR.2.2	T1	A one-template model based on an error-free alignment derived from the structural superposition of the target, T1, and T2.

Description of different types of models used to evaluate the contribution of the second template in structural complementarity in the context of varying alignment quality. T1 and T2 refer to Template1 and Template2.

The relationship between alignment accuracy and model quality improvement due to structural complementarity is also examined i) indirectly by comparing structural complementarity in SEQ and STR models, and ii) directly by evaluating the alignment accuracy of SEQ alignments. Though the latter is the more rigorous comparison, we have deliberately carried out the analysis in both ways as the alignment accuracy is not a directly observable quantity in real modeling cases

Throughout the study model accuracy is measured by root mean squared deviation (RMSD) of the equivalent Cα atoms between the modeled and experimental structure of the target sequence. Since the data set has been designed such that coverage of all targets by the models is always 100%, there is no need to include coverage into the accuracy assessment (see methods for details).

### Two-template vs. one-template model accuracy

**Figure 3:**
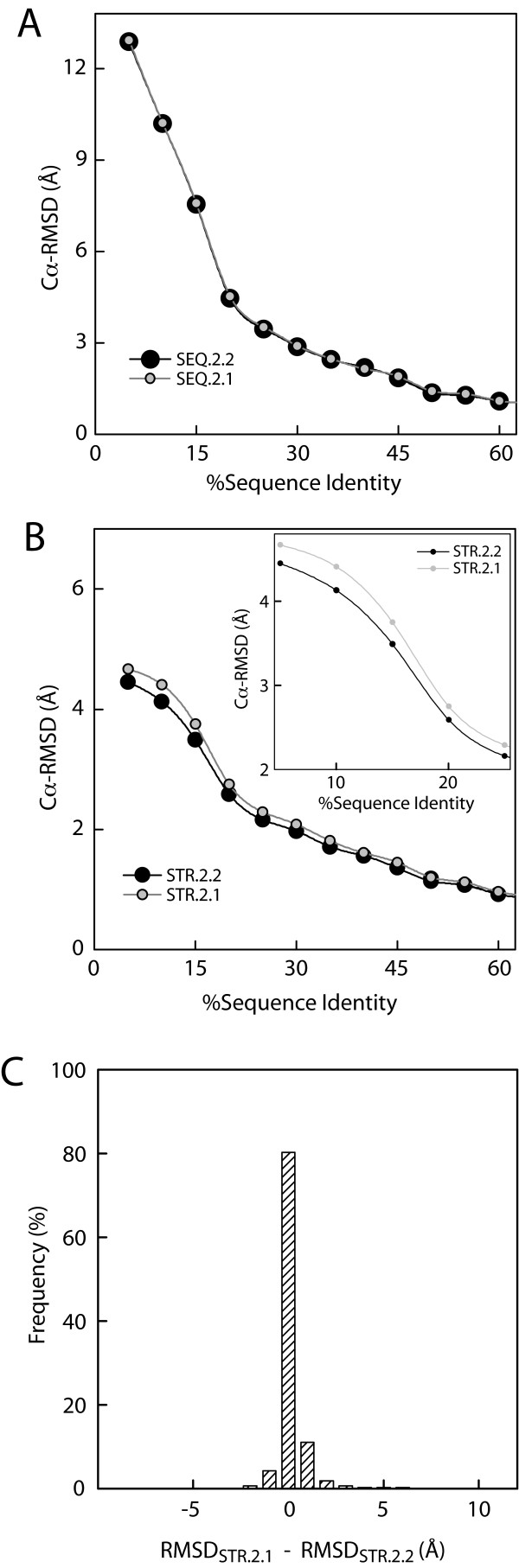
**Accuracy of multiple-template models**. **(A) **Comparison of overall accuracy between single and two-template SEQ models, SEQ.2.2 (large black filled circle) and SEQ.2.1 (small gray circle). **(B) **Comparison between single and two-template STR models, STR.2.2 (filled circle) and STR.2.1 (small gray circle). The lower sequence identity region is highlighted in the inset. Because of the large number of cases analyzed (> 10,000 models per curve) even the small differences shown here are statistically significant based on the Student t test. Thus, for clarity no error bars are shown. **(C) **Distribution of difference in RMSD between one-template (STR.2.1) and two-template models (STR.2.2) built using structure-based alignments (RMSD_STR.2.1 _- RMSD_STR.2.2_). Only models with S1 ≤ 40% are shown here.

To identify accuracy improvements due to structural complementarity the SEQ.2.1 models were analyzed (same modeling alignment of the two-template SEQ.2.2 model but built using only the structure of Template1, see Figure 2). Figure 3A shows that on average over all the cases the difference between SEQ.2.2 and SEQ.2.1 models is negligible. This suggests that, for the simple SEQ alignments, in most cases there is no average gain in model accuracy from structural complementarity. In fact, the distributions of ΔRMSD between SEQ2.1 and SEQ.2.2 models showed that structural complementarity can be observed in only a small fraction of SEQ models (data not shown). While the number of SEQ cases showing structural complementarity is too small to be visible in the average accuracy curves (Figure 3A) it is interesting to determine what factors contribute to it.

### Structural complementarity vs. alignment accuracy

To determine whether the apparent lack of structural complementarity observed in SEQ models is caused by errors in the alignment, two tests were carried out. First, the set of one-template and two-template models based on structural alignments were analyzed (STR2.1 and STR.2.2) to measure structural complementarity in the absence of alignment errors. Second, a direct comparison of alignment accuracy and structural complementarity in SEQ model was carried out. The comparison of STR models shows that two-template models are more accurate than one-template models (Figure 3B). The comparison of the average accuracy of STR.2.2 models with models based on ideal template chimeras, which represent a perfect two-template model (see methods), showed no difference (data not shown) indicating that the fact that the increase in model accuracy is small and is not a consequence of limitations in the modeling approach. The distribution of ΔRMSD between STR.2.1 and STR.2.2 models (Figure 3C) shows that in ~80% of the cases there is no model improvement upon addition of the second template, in a small fraction of cases (~6%) there is minimal deterioration of the models, and in ~14% of cases improvement of the model accuracy is observed. In the most favorable cases this improvement can reach up to 6 Å RMSD (Figure 3C), which is relatively large for changes that are not related to the alignment. As previously mentioned this improvement is a consequence of structural complementarity, thus suggesting that structural complementarity can more readily be observed in the context of highly accurate alignments.

**Figure 4:**
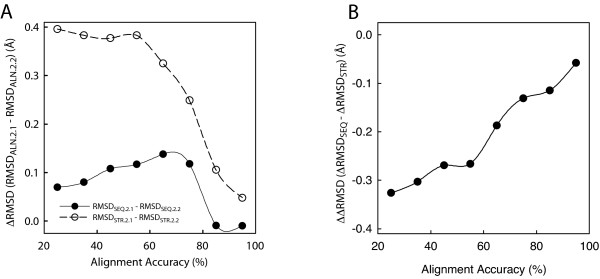
**Relationship between structural complementarity and alignment accuracy**. **(A) **The structural complementarity, ΔRMSD_ALN _= (RMSD_ALN.2.1_-RMSD_ALN.2.2_), of SEQ models (black filled circles) and STR models (empty circles) is shown as a function of SEQ alignment accuracy. The STR curve represents the maximum achievable structural complementarity for each alignment accuracy bin. **(B) **Difference between the observed structural complementarity in SEQ models (ΔRMSD_SEQ_) and maximum achievable structural complementarity (ΔRMSD_STR_) as a function of SEQ alignment accuracy.

To further explore the relationship between structural complementarity and alignment quality, the accuracy of SEQ alignments was measured by comparing them with the corresponding STR alignment (see Methods). A plot of ΔRMSD_ALN _(RMSD_ALN.2.1_-RMSD_ALN.2.2_) as a function of alignment accuracy (Figure 4A) confirms that the amount of structural complementarity is dependent on alignment accuracy. The drop in absolute structural complementarity at very high alignment accuracy (> 75%) is explained by the fact that the maximum possible structural complementarity (the one obtained when the modeling alignment is perfect, i.e. STR alignments) decreases with increasing alignment accuracy since the similarity between the two template structures also increases. This is evidenced by the measurement of ΔRMSD between STR.2.1 and STR.2.2 for the STR models corresponding to every pair of SEQ.2.1 and SEQ.2.2 models within an alignment accuracy bin (Figure 4A, dashed line). The difference between the maximal structural complementarity, ΔRMSD_STR _= (RMSD_STR.2.1_-RMSD_STR.2.2_), and the observed structural complementarity in SEQ models, ΔRMSD_SEQ _= (RMSD_SEQ.2.1_-RMSD_SEQ.2.2_), clearly correlates with alignment accuracy (Figure 4B).

These results, together with those in the previous section, indicate that the positive effect of structural complementarity on the average accuracy of multiple template models can only be obtained when the modeling alignment is highly accurate. The fact that an accurate alignment is necessary to obtain structural complementarity is not surprising. The regions of the templates that are more likely to complement each other are the less conserved regions, which will also contain the most alignment errors. If the complementary regions are not correctly aligned the benefits of the structural information are lost. This same interplay between alignment errors and structural information also affects loop modeling [21], where a good model building protocol may be limited by anchor residues that are inaccurate due to alignment errors. Since insertions tend to occur more frequently in less conserved regions the anchor residues for loop modeling will tend to be aligned less accurately than other regions of the protein. These results once again stress how crucial the alignment quality is in comparative modeling and show that the benefits of a more accurate alignment are amplified in the case of multiple-template modeling by the additional accuracy gains from structural complementarity. Thus, these results suggest that iterative approaches that combine alignment improvement or selection with explicit model building and evaluation may particularly benefit from the use of multiple templates [19,22-24]. The alignment improvement signal would only be strengthened by the additional increase in accuracy due to structural complementarity, once the alignment accuracy reaches a certain level.

### Template combinations resulting in improved model accuracy

**Figure 5:**
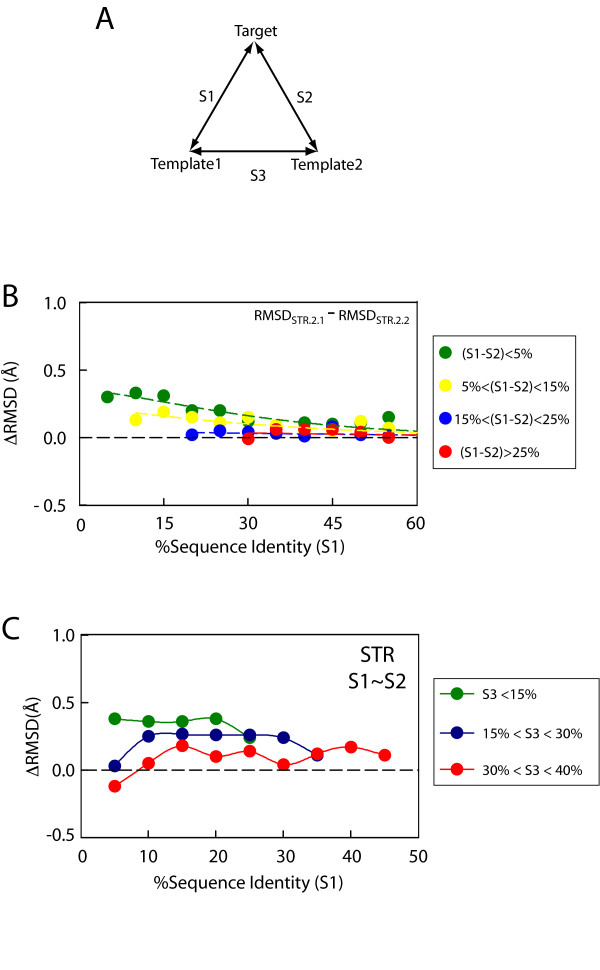
**Effect of the relative Target:Template and Template1:Template2 sequence similarity on two-template model accuracy**. **(A) **Definition of sequence similarities between Target, Template1, and Template2. **(B) **Difference in RMSD for models built using structure-based alignments (RMSD_STR.2.1 _- RMSD_STR.2.2_) as a function of Target:Template1 sequence identity (S1) for different ranges of [S1–S2]. The colored circles green, yellow, blue to red are in increasing order of [S1–S2]. The absence of data at lower sequence identity (for yellow, blue and red) is due to the fact that for large values [S1 – S2], small S1 is not possible. **(C) **Difference in RMSD for models built using structure-based alignments (RMSD_STR.2.1 _- RMSD_STR.2.2_) as a function of Target:Template1 sequence identity (S1) for different ranges of S3. The colored circles, green, blue to red, are in the increasing order of S3. Only models with S1 similar to S2 are shown here. The absence of data points (green and blue) for higher sequence identity is due to the fact that certain combinations of S1, S2, and S3 are not possible.

With the aim of determining if the two-template modeling cases that show the largest accuracy improvement with respect to their one-template counterparts share any common attributes, the model accuracy improvement (ΔRMSD) was measured as a function of the sequence similarity between target and templates (S1 and S2) and the sequence similarity between the two templates (S3) (see Figure 5A). Figure 5B shows the change in model accuracy improvement (ΔRMSD) as a function of the difference between S1 and S2. It is observed that the two-template models (STR.2.2) tend to be more accurate than the one-template models (STR.2.1) when S1 is similar to S2. This effect is only observed at relatively low Target:Template sequence identities (S1 < 35%). However, no deterioration of the models is observed when S1 is very different from S2. Thus, having templates that are equidistant from the target in terms of sequence similarity appears to be beneficial for template complementarity. A trivial way of assuring that S1 is similar to S2 is to select two templates that are very similar to each other (i.e. S3 is close to 100% sequence identity). This of course would not result in an improved two-template model because two templates that are nearly identical will in practice function as a single template (i.e. no complementarity). Hence, the influence of S3 on the accuracy of two-template models needed to be measured in the context of cases where S1 and S2 are very similar (S1 – S2 < 5%). Both in the case of SEQ models and STR models the maximal improvement was observed when S3 is very low (< 15% sequence identity) and the effect slightly decreases as S3 increases (Figures 5C). Hence, when the templates are not only equidistant from the target but also share low sequence similarity between them the accuracy improvement of the two-template model with respect to the one-template model is maximized. One can broadly say that a symmetrical combination (S1 ~ S2 ~ S3) would show a high potential for improvement when S1 is less than 30%.

### Model improvement vs. model deterioration

**Figure 6:**
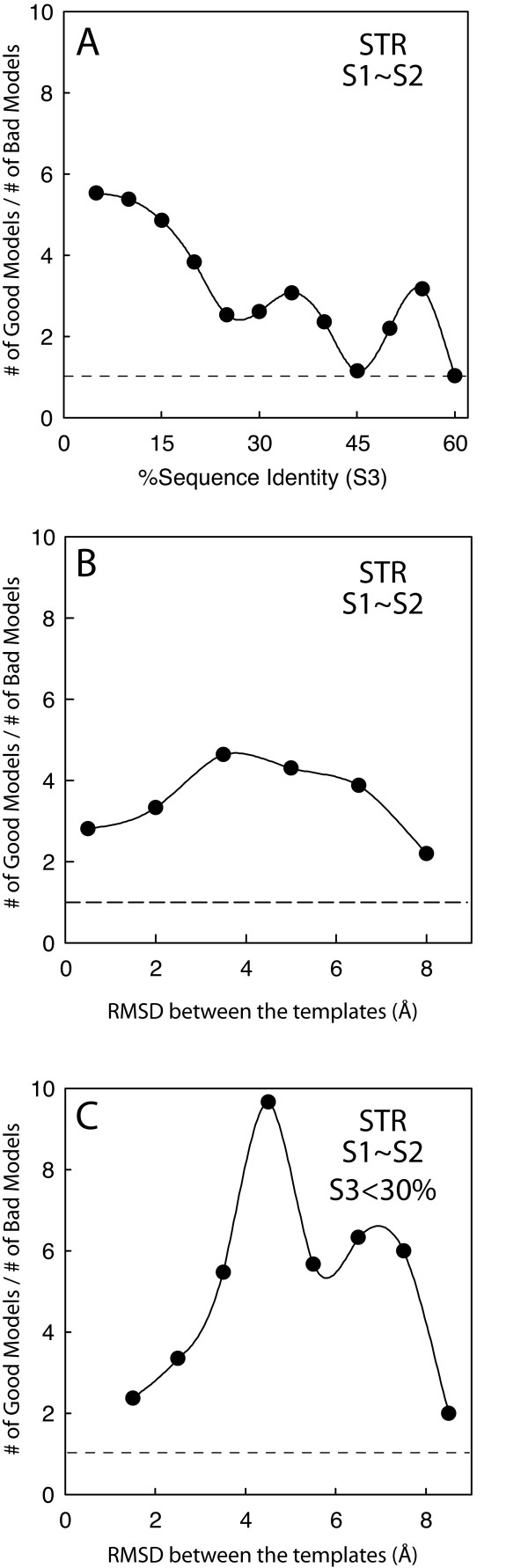
**Proportion of Good/Bad models as a function of S3 and RMSD between the templates**. Accuracy is measured by ΔRMSD defined as (RMSD_STR.2.1 _- RMSD_STR.2.2_). Models are defined as: good: ΔRMSD ≥ 1 Å; bad: ΔRMSD ≤ -1 Å; or neutral: 1 Å > Δ RMSD > -1 Å. In all plots only models based on template combinations for which S1–S2 is less than 5% are included. **(A) **The ratio between the number of Good and Bad STR models as a function of S3, the sequence identity between the templates. **(B) **The good/bad ratio as a function of the RMSD between the two templates. **(C) **The good/bad ratio as a function of the RMSD between the two templates; in these plots the additional restriction of S3 < 30% is imposed on all selected models with the aim of showing the complementarity between S3 and template RMSD selection.

If we define models that improve their accuracy upon addition of the second template by more than 1 Å RMSD as "good" and models that decrease their accuracy by more than 1 Å RMSD as "bad". For a given template selection criterion, we measure the ratio between the number of "good" and "bad" models. Models for which the change in accuracy is less than 1 Å RMSD are ignored. As mentioned above, the sequence similarity between the two templates (S3) is in a way a measure of the potential for complementarity between them. For example, if the two templates share very high similarity, there is little chance that they can complement each other, effectively functioning as a single template. On the other hand, if the two templates are very different from each other it may be difficult to find common alignable elements to transit from the use of one template to the other. Figure 6A shows the change in the ratio of good to bad models as a function of S3. For STR models the ratio of good to bad models shows consistent growth when S3 falls below 30% and reaching values above 5 when S3 is below 15%. A more direct way of measuring the potential complementarity between the templates is to use the structural similarity between them instead of (or in addition to) the sequence similarity S3. Figure 6B shows the change in the ratio of good to bad models as a function of the Cα RMSD between Template1 and Template2. In the range of 2.5 to 6 Å RMSD a ratio of 4 times more good models than bad models (Figure 6B) is observed for STR models. At low sequence identity the correlation between sequence and structural similarity is not strong [1], thus it is possible that S3 and RMSD between the templates are complementary to each other as measures to select optimal template combinations. To explore this possibility the ratio of good to bad models was plotted as a function of the RMSD between the templates, but only for the models where S3 is below 30% (the optimal range from Figure 6A). For STR models a large increase in the ratio was observed (Figure 6C) with values above 5 in the range from 3.5 to 7.5 Å RMSD and a peak value of 10 at 4.5 Å RMSD.

### Selection of optimal template combinations

**Figure 7:**
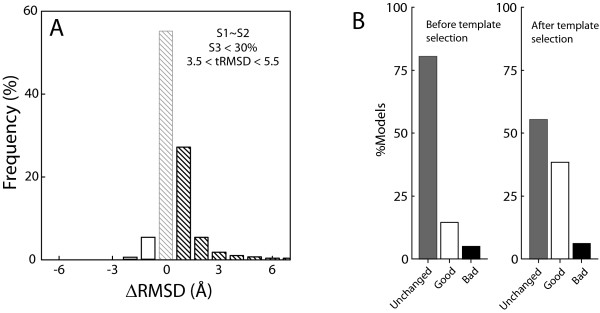
**Distribution of accuracy differences between one-template and two-template models for a selected subset**. **(A) **Difference in RMSD (ΔRMSD) for models built using structure-based alignments (RMSD_STR.2.1 _- RMSD_STR.2.2_). Only models with S1 – S2 less than 5%, S1 < 30%, S3 < 30% and template RMSD between 3.5 and 5.5 Å are shown here. The dark bars correspond to Good models (see figure 6 legend), the empty bars to Bad models, the light bars to Neutral models. **(B) **Fraction of Neutral (unchanged), Good and Bad models in the dataset before and after applying the template selection criteria described above.

The results shown above suggest a strategy for choosing templates for two-template modeling. Template pairs should be selected such that (S1 – S2) < 5%, S3 < 30%, and the RMSD between the templates is in the range of 3.5–5.5 Å. If these selection criteria are applied to our complete set of STR models we observe that 78.1% of the selected two-template models show no significant change with respect to the one-template model, 2.1% show deterioration, and 19.8% show improvement (Figure 7 A and 7B). The ratio of good to bad models improves from 3.5 to 9.4 when compared with the unfiltered set (Figures 3C and 7B). This ratio corresponds to a 90.4% chance of obtaining a more accurate model vs. obtaining a less accurate one. These template selection rules can be particularly useful in the context of large-scale automated modeling [1,17] and in the context of structural genomics target ranking [25]. As structural genomics aims to provide enough experimental protein structures to accurately model the remaining proteins by comparative modeling it will be important to include considerations about the effect that structural complementarity of different structural genomics targets will have on the accuracy of the resulting models. The selection rule can help to guide the choice of the next template for structure determination in a family of domains. For example, in a hypothetical protein family with 100 members out of which only 3 have known structures, the choice of the member out of the remaining 97 sequences whose structure is to be determined next can be guided by the S1, S2 and S3 relationship, such that the structure of the 4^th ^member will enhance the modeling accuracy of most of the remaining 96 members by using multiple template models.

**Figure 8:**
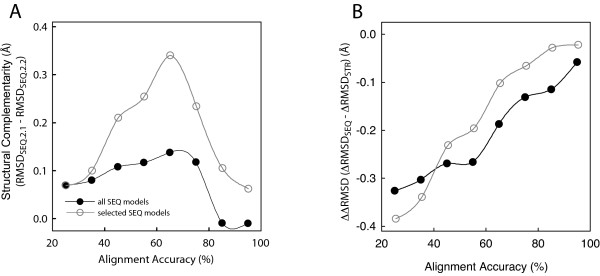
**Relationship between structural complementarity and alignment accuracy in the selected subset**. The selected models correspond to those described in Figure 7. **(A) **The structural complementarity, ΔRMSD_SEQ _= (RMSD_SEQ.2.1_-RMSD_SEQ.2.2_), of selected SEQ models (empty circles) is shown as a function of SEQ alignment accuracy. The curve for all SEQ models from Figure 4A (black circles) is shown for comparison. **(B) **Difference between observed structural complementarity in SEQ models (ΔRMSD_SEQ_) and maximum achievable structural complementarity (ΔRMSD_STR_) as a function of SEQ alignment accuracy is shown for the selected models (empty circles) and for all models (black circles).

It is encouraging to notice that template combinations that fall into the "preferred" range (S1 ≅ S2, S1 < 30%, S3 < 30%, Template RMSD 3.5–5.5 Å) show a high probability of resulting in improved model accuracy. In the case of sequence-based alignments this benefit is less pronounced because of the low alignment accuracy. However, in structure-based alignments the effect of selecting optimal template combinations is significant and suggests that if accurate alignments can be achieved the model accuracy gains from template complementarity can be substantial. This is further supported by revisiting the relationship between structural complementarity and alignment accuracy for this subset of optimal template combinations (Figure 8). The absolute structural complementarity obtained at equivalent alignment accuracies is higher in the optimal subset set as compared to the complete set (Figure 8A) as a consequence of the higher potential for complementarity of the optimal subset. The overall relationship between maximal structural complementarity ΔRMSD_STR _= (RMSD_STR.2.1_-RMSD_STR.2.2_), and the observed structural complementarity in SEQ models, ΔRMSD_SEQ _= (RMSD_SEQ.2.1_-RMSD_SEQ.2.2_), is not only maintained in the optimal subset (Figure 8B) but shows a more rapid tendency to improve model quality with increase in alignment accuracy. The selection of optimal template combinations in the STR case is quite robust, with only a very small chance of deteriorating the model as a consequence of adding a second template. The maximum accuracy gains are obtained at low Target:Template sequence identities which is where most of the modeling cases fall [1]. This is also the range where the most effort in model improvement is required [5], and where the models are most informative compared to their templates [6]. Thus, given that Target:Template alignment methods continue to improve, it is expected that the benefit of structural complementarity in multiple template modeling will also increase. The magnitude of the potential improvements (2–6 Å RMSD) is quite significant compared to what the best refinement methods can obtain in some selected cases [12,13].

## Conclusion

The results of this large-scale (~30,000 models) comprehensive analysis of multiple-template models explain the previous contradictory examples of improvement and deterioration of model quality on inclusion of additional templates [17-19]. Both situations are possible. Combinations of templates with S1 ≅ S2, S1 < 30%, S3 < 30%, and Template RMSD 3.5–5.5 Å show a high probability of improved model accuracy over the single-template model, while most remaining combinations tend to deteriorate the model. Since most modeling cases fall in the sequence identity range below 30%, our results enable judicious choice of additional templates (based on S2, S3 and RMSD between templates) to improve model accuracy. While structural complementarity does not contribute significantly to the average accuracy of simple SEQ models, its role increases as the accuracy of the modeling alignment increases as illustrated by the high accuracy SEQ alignments and the STR alignments. Since template selection is a fundamental step in comparative modeling, and the selection criteria described here are independent of the model building strategy used, the results of our analysis are relevant to any multiple-template modeling case irrespective of the software used. Furthermore, the pre-screening of templates with increased potential for complementarity could prove beneficial in the context of modeling methods that attempt to identify good template combinations through model evaluation by decreasing the size of the search space [20]. The potential improvements obtained from a judicious template selection are also complementary to other approaches for improving model accuracy such as loop modeling [21] and general refinement [12,13]. Because our study is limited to two-template models and fixed alignments it is not representative of the expected model accuracy improvements that could be obtained by using larger numbers of templates and applying simultaneous alignment optimization. However, our results provide a clean description of the underlying relationships between alignment accuracy, template similarity, and model accuracy.

## Methods

### Construction of the data set

Single-domain chains (size: 100–200 residues) of high resolution (2.5 Å or better) X-ray structures were selected from the Protein Data Bank (PDB) [26] using domain definitions from CATH [27]. Chains were grouped according to structural classes (i.e. all-α, all-β and α/β). Only the all-β and α/β fold-class proteins were used for the complete analysis. All-α proteins showed the same trends and differences between one-template and two-template models as all-β and α/β proteins, but with different average accuracies. For simplicity, they were eliminated from the rest of the analysis although the same conclusions apply to them. Redundancy in the set was eliminated by grouping together chains from the same homologous superfamily (same value of the first four CATH levels) that shared a sequence similarity of at least 95% identity over more than 85% the sequence length. Only the highest resolution member of each of these groups was retained as a representative in the final set. Homologous superfamilies with at least three representative chains were considered for the following steps. The representative chains within the same homologous superfamily were structurally aligned with each other using program CE [28]. A combination of three chains (a triplet) was selected if at least two out of the three inter-chain structural alignments had a CE Z-score higher than 4.5. A total of 145 homologous superfamilies satisfied these criteria. A total of 10,641 chains triplets were chosen from these families such that bias from larger families was below a predefined cutoff. Entropy of the dataset was used to set the cutoff. Within each triplet only the common region of the structures (based on the CE alignments) was selected, hence any Target:Template combination within the triplet produces 100% coverage of the target.

Since each chain of a triplet can be the target with the other two as templates, the total number of models for the dataset is 31,923 (3 × 10,641). The sequence identity assigned to a particular **Target**:**Template1**:**Template2 **triplet was that of the Target:Template pair with the higher sequence identity. Template1 always refers to the template with the higher sequence identity.

### Sequence identity measure

Percent of sequence identity was used as the measure of sequence similarity because it is the most common variable used to describe comparative models making it convenient for comparing our results with previous work addressing the accuracy of comparative models [4-6]. Sequence identity was defined as the ratio between the number of identical aligned residue pairs and the number of target residues in the Target:Template alignment. For Template1:Template2 comparisons the sequence identity was defined as the as the ratio between the number of identical aligned residue pairs and the number of residues in the shorter of the two sequences. The following notations for a Target:Template1:Template2 two-template model are used throughout the text (see also Figure 5A):

(i) **S1 **= Target:Template1 sequence identity

(ii) **S2 **= Target:Template2 sequence identity

(iii) **S3 **= Template1:Template2 sequence identity

By definition S1 ≥ S_2_. Thus, the corresponding single-template model of the target is based on Template1 and is referred to as Target:Template1. In the results, sequence identity S1 is used as the reference sequence identity for both the two-template model as well as the corresponding single-template model.

### Model notation

A three-character (ALN.*X.Y*) notation is used to describe the models. ALN (SEQ or STR) refers to the method used to build the Target:Template alignment: pairwise SEQuence-alignment or STRucture-alignment. *X *refers to the number of templates used to obtain the alignment and *Y *refers to the number of the templates used in model building process (Figure 2). For example, SEQ.2.1 refers to a model based on a pairwise sequence alignment where the alignment is obtained using two templates but the model is built using the structure of Template1 only. The various models studied here are SEQ.2.2, SEQ.2.1, STR.2.2, and STR.2.1. Where 2.2 models correspond to typical two-template models and 2.1 models correspond to one-template models with identical alignments to the 2.2 models. These 2.1 models are used to study the contribution of structural complementarity on the final accuracy of the two-template models in the absence of any alignment effect (see below and Figure 2).

### Target:Template alignment and model building

Models were calculated using the alignments described below and the template structures as input to the default 'model' routine of program MODELLER version 6v2 [29]. **SEQ.2.2**: The structural alignment between the two templates (Template1, Template2) was first generated using the ALIGN3D command of MODELLER. The target sequence was aligned to this structural alignment of the templates, using the ALIGN command of MODELLER, without modifying the structural alignment (Figure 2). **SEQ.2.1**: The sequence of Template2 is eliminated from SEQ.2.2 (See Figure 2).**STR.2.2**: Structural alignment between the three structures (Target, Template1, and Template2) was generated using the ALIGN3D command of MODELLER.**STR.2.1**: The sequence of Template2 is eliminated from STR.2.2.

### Alignment Accuracy Measurement

Alignment accuracy was measured as defined by Sauder et al. [30], namely, the ratio between the number of correctly aligned residue pairs and the total aligned residue pairs in a given alignment. A residue pair is defined as correctly aligned if it is the same in the reference ("error-free") alignment. STR.2.2 alignments were used at the error-free reference.

### Construction of Template Chimera

Idealized template chimeras for particular Target:Template1:Template2 combinations were constructed by selecting the structurally closest equivalent segment (for each non overlapping target segments) from either of the two templates. The combination of these "best" segments from each of the templates results in an ideal template chimera that can be used to evaluate the efficiency with which the modeling program (i.e. MODELLER) combines the information from both templates. Closeness among the equivalent residues is determined by measuring the distance between the target residue backbone atoms and that of the equivalent template residue after optimal pairwise structural superposition of the target with each of the templates.

### Overall Accuracy Measurement

Overall accuracy was measured by computing the root mean square deviation (RMSD) between the equivalent Cα atoms in the optimal superposition of target and model structures as it is the most common evaluation performed systematically for comparative models [5,31,32]. Since the sequences of target and model are identical, a sequence-based alignment was used to guide the initial structural superposition. Equivalent atoms are defined as those that are within 3.5 Å of their corresponding atom in the target after superposition of the structures. Superposition of structures is carried out by minimizing the RMSD between the equivalent Cα atoms. However, all accuracy measurements refer to the RMSD of all Cα atoms irrespective of cutoff. All calculations are implemented in the SUPERPOSE command of program MODELLER. As the structural differences between main-chains of models obtained from various comparative modeling programs are very small [4], results of the current analysis are based only on a single modeling program, MODELLER [29]. In addition, as there are differences in the quality of side-chain modeling in different comparative modeling programs [33], the present accuracy analysis is restricted to comparison of backbone structures on which the template structure has a larger influence than on the side-chains [18].

## Authors' contributions

SC built the dataset, created the analysis procedure, collected and analyzed the data, and contributed to the design of the study. SG contributed to the creation of specialized alignments. BZ and SB build the first version of the dataset and carried out initial experiments with multiple template modeling that helped shape the project. RS conceived the study, guided the project, and contributed to the design. SC and RS wrote the manuscript.
